# A Framework for Human-Robot-Human Physical Interaction Based on N-Player Game Theory

**DOI:** 10.3390/s20175005

**Published:** 2020-09-03

**Authors:** Rui Zou, Yubin Liu, Jie Zhao, Hegao Cai

**Affiliations:** State Key Laboratory of Robotics and Systems, Harbin Institute of Technology, Harbin 150001, China; 18b908014@stu.hit.edu.cn (R.Z.); jzhao@hit.edu.cn (J.Z.); hgcai@hit.edu.cn (H.C.)

**Keywords:** physical human-robot interaction, game theory, adaptive optimal control, robot control

## Abstract

In order to analyze the complex interactive behaviors between the robot and two humans, this paper presents an adaptive optimal control framework for human-robot-human physical interaction. N-player linear quadratic differential game theory is used to describe the system under study. N-player differential game theory can not be used directly in actual scenerie, since the robot cannot know humans’ control objectives in advance. In order to let the robot know humans’ control objectives, the paper presents an online estimation method to identify unknown humans’ control objectives based on the recursive least squares algorithm. The Nash equilibrium solution of human-robot-human interaction is obtained by solving the coupled Riccati equation. Adaptive optimal control can be achieved during the human-robot-human physical interaction. The effectiveness of the proposed method is demonstrated by rigorous theoretical analysis and simulations. The simulation results show that the proposed controller can achieve adaptive optimal control during the interaction between the robot and two humans. Compared with the LQR controller, the proposed controller has more superior performance.

## 1. Introduction

In the past decade, physical human-robot interaction has attracted the attention of the research community due to the urgent requirement for robot technology in unstructured environment [1,2,3,4]. Physical human-robot interaction combines the advantages of humans and robots, which means that humans are good at reasoning and problem solving with high flexibility, while robots perform well in terms of execution as well as guaranteeing the accuracy of task execution [5,6]. The combination of these advantages has led to the wide application of physical human-robot interaction, such as teleoperation [7,8], collaborative assembly [9,10], and collaborative transportation [11,12,13].

Two types of specific human robot interaction strategies have been widely studied: co-activity type of interaction strategy and master-slave control strategy [14,15]. Co-activity type of interaction strategy is used in typical rehabilitation robots that help limb movement training or intelligent industrial systems that support heavy objects to resist gravity, where robots completely ignore human users’ behaviors [16,17]. In contrast, the master–slave control strategy is used in the teleoperated robots or force extender exoskeletons use where robots completely follow the control of human users [18]. However, these strategies can only be used for specific interactive behaviors, the general framework for analyzing various interactive behaviors between robot and humans is still missing [19,20].

It has been pointed out that game theory can be used as a general framework to analyze complex interactive behaviors between multiple agents because different combinations of individual cost functions and different optimization objectives can be used to describe various interactive behaviors in game theory [21]. In [22], the human and the robot were been regarded as two agents and game theory was used in order to analyze the performance of the two agents. In [23], the optimal control was obtained for a given game with a linear system cost function by solving the coupled Riccati equation. In [24], an optimal control algorithm was developed for human-robot collaboration by solving the Riccati equation in each loop. In [25,26,27,28], policy iteration was used to solve the Nash equilibrium solution in order to improve the calculation speed. In [29], cyber-physical human systems was modeled via an interplay between reinforcement learning and game theory. In [30], haptic shared control for human-robot collaboration was modeled by a game-theoretical approach. In [31], human-like motion planning was studied based on game theoretic decision making. In [32], cooperative game was used for human-robot collaborative manufacturing. In [33], a bayesian framework was proposed for nash equilibrium inference in human-robot parallel play. In [19], non-cooperative differential game theory was used to model human-robot interaction system that results in a variety of interaction strategies. However, the above studies only consider two agents, that is, the interaction between one human and one robot. Therefore, the aforementioned methods are not suitable for human-robot-human physical interaction where more than one human interact with one robot physically. It is worth noting that the physical interaction between one robot and two humans will bring greater advantages such as operating larger loads, improving the flexibility and robustness of the system [28,34,35,36,37]. These greater advantages are brought by the team collaboration between the robot and two humans. To the authors’ acknowledgment, no literature have researched the problem of the physical interaction between one robot and two humans based on game theory.

In the paper, a general adaptive optimal control framework for human-robot-human physical interaction is proposed based on N-player game theory. Accordingly, the robot and two humans can interact with each other optimally by learning each other’s control. N-player differential game theory was used to model the human-robot-human interaction system in order to analyze the complex interactive behaviors between the robot and two humans. In N-player differential game theory, humans’ control objectives are assumed to be knowledge [38,39]. However, N-player differential game theory can not be used directly in actual scenerie since the robot cannot know humans’ control objectives in advance. In order to let the robot know humans’ control objectives, the paper presents an online estimation method to identify unknown humans’ control objectives based on the recursive least squares algorithm. Subsequently, the Nash equilibrium solution of the multi-human robot physical interaction is obtained by solving the coupled Riccati equation to achieve coupled optimization. Finally, the effectiveness of the proposed method is demonstrated by rigorous theoretical analysis and simulation experiments. This paper makes the following four contributions.

(1) N-player differential game theory is firstly used to model the human-robot-human interaction system.
(2) An online estimation method to identify unknown humans’ control objectives based on the recursive least squares algorithm is presented.
(3) A general adaptive optimal control framework for human-robot-human physical interaction is propose based on (1) and (2).
(4) The effectiveness of the proposed method is demonstrated by rigorous theoretical analysis and simulation experiments.

The remainder of this paper is organized, as follows: Section 2 models the human-robot-human physical interaction system based on N-player differential game theory. Section 3 establishes an adaptive optimal control law, and the control performance of the system is analyzed theoretically. Section 4 verifies the effectiveness of the proposed method through simulation experiments. Finally, Section 5 concludes this work.

## 2. Problem Formulation

### 2.1. System Description

**Figure 1:**
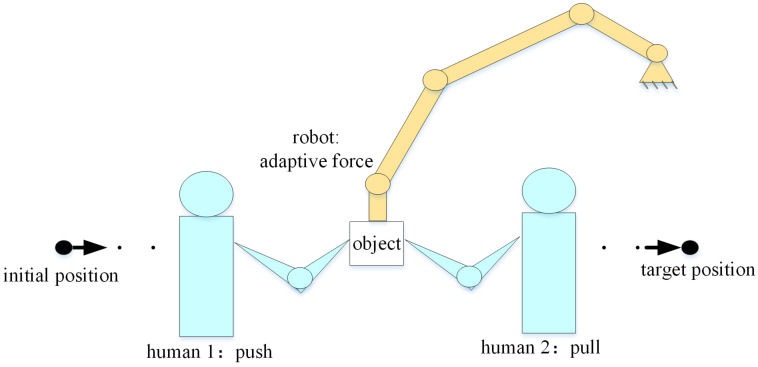
A scenario where the humans and the robot collaborate to perform an object transporting task.

The system considered contains two humans and one robot. An example scenario is shown in Figure 1, where the robot and the humans collaborate to perform an object transporting task. In this shared control task, when the control objectives of humans’ change, the robot should recognize the humans’ control objectives and response adaptively and optimally. The forces exerted by the humans on the object are measured by force sensors at the interaction point. It is worth noting that the humans’ control objectives are unknown to the robot.

The forward kinematics of the robot are described as

(1) x(t)=ϕ(q(t))

where $x\left(t\right)\in R^{m}$ and $q\left(t\right)\in R^{n}$ are the positions in Cartesian space and joint space respectively, *m* and *n* are degrees of freedom. Derivation of Equation (1) with time can be obtained

(2) $$\dot{x}\left(t\right)=J\left(q\left(t\right)\right)\dot{q}\left(t\right)$$

where $J\left(q\left(t\right)\right)\in R^{m\times n}$ is the Jacobian matrix.

The following impedance model is given in Cartesian space

(3) $$M_{d}\ddot{x}\left(t\right)+C_{d}\dot{x}\left(t\right)=u\left(t\right)+f_{1}\left(t\right)+f_{2}\left(t\right)$$

where $M_{d}\in R^{m\times m}$ is the desired inertial matrix, $C_{d}\in R^{m\times m}$ is the damping matrix, $u\left(t\right)\in R^{m}$ is the control input in the Cartesian space [40,41,42], $f_{1}\left(t\right)\in R^{n}$ is the contact force between object and human 1, $f_{2}\left(t\right)\in R^{n}$ is the contact force between object and human 2.

To track a common and fixed target $x_{d}\in R^{m}$ (${\dot{x}}_{d}\in R^{m}$) in cooperative object transporting task, Equation (3) can be transformed, as following

(4) $$M_{d}\left(\ddot{x}\left(t\right)-{\ddot{x}}_{d}\right)+C_{d}\left(\dot{x}\left(t\right)-{\dot{x}}_{d}\right)=u\left(t\right)+f_{1}\left(t\right)+f_{2}\left(t\right).$$

In order to ease the design of the control, Equation (4) can be rewritten as the following state-space form

(5) $$\begin{array}{c} \dot{z}=Az+B_{1}u+B_{2}f_{1}+B_{3}f_{2}\\ z=\left[\begin{array}{c} x\left(t\right)-x_{d}\\ \dot{x}\left(t\right) \end{array}\right],A=\left[\begin{array}{cc} 0_{m} & 1_{m}\\ 0_{m} & -M_{d}^{-1}C_{d} \end{array}\right]\\ B_{1}=B_{2}=B_{3}=B=\left[\begin{array}{c} 0_{m}\\ M_{d}^{-1} \end{array}\right] \end{array}$$

where $0_{m}$ and $1_{m}$ denote m×m zero and unit matrices, respectively.

### 2.2. Problem Formulation

According to non-cooperative differential game theory, in the paper, the interaction between the robot and the humans is described as a game between N players (in this paper, N=3) [43]. In the game, each player will minimize their respective cost function

(6) $$\begin{array}{cc} \Gamma & \equiv \int_{t_{0}}^{\infty }z^{T}Qz+u^{T}ud_{t}\\ \Gamma_{1} & \equiv \int_{t_{0}}^{\infty }z^{T}Q_{1}z+f_{1}^{T}f_{1}d_{t}\\ \Gamma_{2} & \equiv \int_{t_{0}}^{\infty }z^{T}Q_{2}z+f_{2}^{T}f_{2}d_{t}\\ Q & =\left[\begin{array}{cc} Q_{01} & 0_{n\times n}\\ 0_{n\times n} & Q_{02} \end{array}\right]\\ Q_{1} & =\left[\begin{array}{cc} Q_{11} & 0_{n\times n}\\ 0_{n\times n} & Q_{12} \end{array}\right]\\ Q_{2} & =\left[\begin{array}{cc} Q_{21} & 0_{n\times n}\\ 0_{n\times n} & Q_{22} \end{array}\right] \end{array}$$

where $\Gamma ,\Gamma_{1},\Gamma_{2}$ are cost functions of the robot, human 1, and human 2, respectively, $Q,Q_{1},Q_{2}$ are state weights matrices of the robot, human 1 and human 2, respectively. Each player achieves the cooperative object transporting task by minimizing the error to the target while minimizing their own costs. $Q,Q_{1},Q_{2}$ contain two components corresponding to position regulation and velocity, respectively. $Q_{01},Q_{11},Q_{21}$ correspond to position regulation and $Q_{02},Q_{12},Q_{22}$ correspond to velocity.

In [27], the N-player game has been studied if the cost functions are known. However, $\Gamma_{1},\Gamma_{2}$ are unknown to the robot because they are determined by the humans. Therefore, a method is proposed in the paper to estimate $\Gamma_{1},\Gamma_{2}$ in order to achieve adaptive optimal control and, thus, the human-robot-human cooperative object transporting task.

### 2.3. N-Player Differential Game Theory

Based on the differential game theory of linear systems, for *N*-player game the following linear differential equation [43] is considered:

(7) $$\dot{z}=Az+B_{1}u_{1}+\cdots +B_{N}u_{N},z\left(0\right)=z_{0}.$$

Each player has a quadratic cost function that they want to minimize:

(8) $$\Gamma_{i}=\int_{0}^{\infty }z^{T}Q_{i}z+u_{i}^{T}u_{i}d_{t},i=1,\cdots ,N$$

Different types of multi-agent behaviors are defined in game theory, which can be achieved through different concepts of game equilibrium [44,45]. In this paper, Nash equilibrium is considered. In the sense of Nash equilibrium, each player minimizes their cost function:

(9) $$\begin{array}{cc} \; & u_{i}=-\eta_{i}z,\eta_{i}=B_{i}^{T}P_{i}\\ {\left(A-\sum_{j\neq i}^{N}B_{i}\eta_{i}\right)}^{T}P_{i}+P_{i}(A- & \sum_{j\neq i}^{N}B_{i}\eta_{i}{)}_{i}+Q_{i}-P_{i}B_{i}B_{i}^{T}P=0,i=1,\cdots ,N \end{array}$$

where *N* is equal to 3 in this paper. In the sense of Nash equilibrium, the humans and the robot minimizes their own cost function:

(10a) $$\begin{array}{cc} u & =-\alpha z\\ \alpha & =B^{T}P_{r} \end{array}$$

(10b) $$\begin{array}{cc} f_{1} & =-\beta z\\ \beta & =B^{T}P_{1} \end{array}$$

(10c) $$\begin{array}{cc} f_{2} & =-\gamma z\\ \gamma & =B^{T}P_{1} \end{array}$$

(10d) $$\begin{array}{c} A_{r}^{T}P_{r}+P_{r}A_{r}+Q-P_{r}BB^{T}P_{r}=0_{2n},A_{r}=A-B\beta -B\gamma \end{array}$$

(10e) $$\begin{array}{c} A_{1}^{T}P_{1}+P_{1}A_{1}+Q-P_{1}BB^{T}P_{1}=0_{2n},A_{1}=A-B\alpha -B\gamma \end{array}$$

(10f) $$\begin{array}{c} A_{2}^{T}P_{2}+P_{2}A_{2}+Q-P_{2}BB^{T}P_{2}=0_{2n},A_{2}=A-B\alpha -B\beta \end{array}$$

where $\alpha \equiv \left[\begin{array}{c} \alpha_{e},\alpha_{v} \end{array}\right]$ is the feedback gain of the robot, $\beta \equiv \left[\begin{array}{c} \beta_{e},\beta_{v} \end{array}\right]$ is the feedback gain of the human 1, $\gamma \equiv \left[\begin{array}{c} \gamma_{e},\gamma_{v} \end{array}\right]$ is the feedback gain of of the human 2. $\alpha_{e},\beta_{e},\gamma_{e}$ are the position error gains, $\alpha_{v},\beta_{v},\gamma_{v}$ are the velocity gains, $P_{r},P_{1},P_{2}$ are the solutions of the above well-known Riccati equation consisting of Equation (10d–f). The robot and the humans influence each other through $A_{r},A_{1}$, and $A_{2}$ in order to achieve the interactive control and the coupling optimization.

β,γ are unknown to the robot. Therefore, we aim to propose a method to estimate them in the following section.

## 3. Adaptive Optimal Control

A recursive least squares algorithm with forgetting factors is used in this paper to get the estimate $\hat{\beta },\hat{\gamma }$ of β,γ in order to estimate the feedback gains of the humans in real time and avoid the data saturation phenomenon caused by the standard least squares algorithm [46]. Subsequently, the estimate ${\hat{Q}}_{1},{\hat{Q}}_{2}$ of $Q_{1},Q_{2}$ can be obtained using Equation (10e,f).

Equation (10e) is used as the model for identification. For convenience, we let $\theta_{1}=-\beta^{T}$, $y_{1}=f_{1}^{T}$, $W=z^{T}$. Subsequently, Equation (10b) can be rewritten as

(11) $$y_{1}=W\theta_{1}.$$

The feedback gain of the human 1 are estimated by minimizing the total prediction error

(12) $$J_{1}=\int_{0}^{t}exp\left(-\lambda_{1}t\right){∥y_{1}\left(s\right)-W\left(s\right){\hat{\theta }}_{1}∥}^{2}ds$$

where $\lambda_{1}$ is the constant forgetting factor. The update rule of the parameter $\theta_{1}$ can be obtained as

(13) $$\begin{array}{cc} {\dot{\hat{\theta }}}_{1} & =-PW^{T}e_{1}\\ \dot{P} & =\lambda_{1}P-PW^{T}WP\\ e_{1} & ={\hat{y}}_{1}-y_{1}. \end{array}$$

The estimated error of ${\hat{\theta }}_{1}$ is

(14) $$e_{\theta_{1}}\left(t\right)=exp\left(-\lambda_{1}t\right)P\left(t\right)P^{-1}\left(0\right)e_{\theta_{1}}\left(0\right).$$

Thus, the estimate $\hat{\beta }$ can be obtained as

(15) $$\hat{\beta }=-{\hat{\theta }}_{1}^{T}.$$

Similarly, we let $\theta_{2}=-\gamma^{T}$, $y_{2}=f_{2}^{T}$, $W=z^{T}$. Afterwards, Equation (10c) can be rewritten as

(16) $$y_{2}=W\theta_{2}.$$

The feedback gain of the human 2 are estimated by minimizing the total prediction error

(17) $$J_{2}=\int_{0}^{t}exp\left(-\lambda_{2}t\right){∥y_{2}\left(s\right)-W\left(s\right){\hat{\theta }}_{2}∥}^{2}ds$$

where $\lambda_{2}$ is the constant forgetting factor. The update rule of the parameter $\theta_{2}$ can be obtained as

(18) $$\begin{array}{cc} {\dot{\hat{\theta }}}_{2} & =-PW^{T}e_{2}\\ \dot{P} & =\lambda_{2}P-PW^{T}WP\\ e_{1} & ={\hat{y}}_{2}-y_{2}. \end{array}$$

The estimated error of ${\hat{\theta }}_{2}$ is

(19) $$e_{\theta_{2}}\left(t\right)=exp\left(-\lambda_{2}t\right)P\left(t\right)P^{-1}\left(0\right)e_{\theta_{2}}\left(0\right).$$

Thus, the estimate $\hat{\gamma }$ can be obtained as

(20) $$\hat{\gamma }=-{\hat{\theta }}_{2}^{T}.$$

Equations (13), (15), (18) and (20) are critical, because they enable each agent to recognize their partners’ control objectives and use Equation (10a–f) to adjust their own control.

In order to ensure the performance of cooperative object transporting task, we let

(21) $$Q+Q_{1}+Q_{2}\equiv C$$

where *C* is the total weight. The cooperative object transporting task fixes the task performance through the total weight C and uses Equation (21) to share the the effort between 2 humans and the robot. Equation (21) makes the proposed controller be able to adjust the contributions between the humans and the robot and makes the humans and the robot take complementary roles as well.

**Figure 2:**
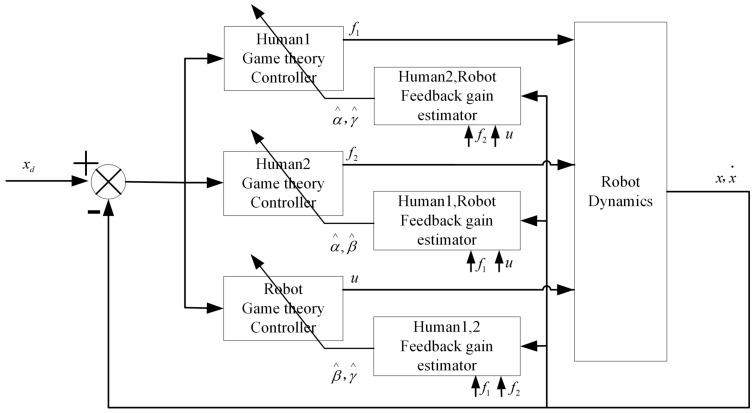
Control Architecture.

The control architecture is shown in Figure 2.

A pseudo-code summarizes the implementation procedures of the proposed method as Algorithm 1.
**Algorithm 1** Adaptive optimal control algorithm based on N-player game**Input:** Current state *z*, target $x_{d}$.**Output:** Robot’s control input *u*, estimated the humans’ cost function state weight ${\hat{Q}}_{1},{\hat{Q}}_{2}$ in Equation (10e,f).**Begin**  Define $x_{d}$, initialize $Q,{\hat{Q}}_{1},{\hat{Q}}_{2},u,f_{1},f_{2},\hat{z},\alpha ,\hat{\beta },\hat{\gamma },P_{r},{\hat{P}}_{1},{\hat{P}}_{2}$, set $\lambda_{1}$ in Equation (13), $\lambda_{2}$ in Equation (18), *C* in  Equation (21), the terminal time $t_{f}$ of one trial.  **While** $t<t_{f}$ do    Measure the position x(t), velocity $\dot{x}\left(t\right)$, and form *z*.    Update $\hat{\beta }$ using Equations (13) and (15), Update $\hat{\gamma }$ using Equations (18) and (20).    Solve the Riccati equation in Equation (10d) to obtain *P*, and calculate the robot’s control input *u*.    Calculate estimated the humans’ cost function state weights ${\hat{Q}}_{1},{\hat{Q}}_{2}$ in Equation (10e,f) using the Riccati equation.    Compute robot’s cost function state weight *Q* according to Equation (21).

**Theorem** **1.**  *Consider the robot dynamics shown in Equation (5). If the robot and the humans estimate the parameters of their partners’ controller and adjust their own control according to Equations (10a–f), (13), (15), (18), (20) and (21), then the following conclusions will be drawn:*

- *The closed-loop system is stable, and $z,\alpha ,\hat{\beta },\hat{\gamma },u$ are bounded.*
- *$\lim_{x\to \infty }{\hat{Q}}_{1}=Q_{1}$, $\lim_{x\to \infty }{\hat{Q}}_{2}=Q_{2}$, which indicate that ${\hat{Q}}_{1},{\hat{Q}}_{2}$ converge to the correct values $Q_{1},Q_{2}$, if z is persistently exciting.*
- *The Nash equilibrium is achieved for th human-robot-human interaction system.*

**Proof of Theorem 1.**  $\hat{\beta },\hat{\gamma }$ influence $u,f_{1},f_{2},z$ as following:

(22) $$\dot{\hat{z}}=A\hat{z}+B\hat{u}+Bf_{1}+B_{2}.$$

By subtracting Equation (5) from Equation (22), we have

(23) $${\dot{e}}_{z}=Ae_{z}+B\left(\hat{u}-u\right)+Be_{f_{1}}+Be_{f_{2}}$$

where $e_{z}=\hat{z}-z$. By considering Equation (10a–c), we have

(24) $${\dot{e}}_{z}=\left(A-B\alpha \right)e_{z}+Be_{\theta_{1}}^{T}z+Be_{\theta_{2}}^{T}z.$$

Consider the Lyapunov function candidate as following

(25) $$W=\frac{1}{2}z^{T}z+\frac{1}{2}e_{\theta_{1}}^{T}e_{\theta_{1}}+\frac{1}{2}e_{\theta_{2}}^{T}e_{\theta_{2}}+\frac{\chi }{2}e_{z}^{T}e_{z}$$

where $\chi =min(\frac{2(\lambda_{1}-\rho )\pi }{\varphi^{2}{∥B∥}^{2}},\frac{2(\lambda_{2}-\rho )\pi }{\varphi^{2}{∥B∥}^{2}})$, with ρ being the upper bound of the maximum eigenvalue of $\dot{P}P^{-1}$, π being the lower bound of the minimum eigenvalue of Bα−A, φ being the upper bound of ∥z∥.

When considering function $V=\frac{1}{2}z^{T}z$ and differentiating *V* with respect to time, we obtain

(26) $$\begin{array}{cc} \dot{V} & =z^{T}\dot{z}=-z^{T}\left(B\alpha +B\beta +B\gamma -A\right)z. \end{array}$$

According to Equation (10d), Bα+Bβ+Bγ−A is positive definite if *Q* is positive definite, it follows $\lim_{t\to \infty }∥z∥=0$. Therefore, *z* is bounded and we define φ as the upper bound of ∥z∥. By differentiating Equation (25), with respect to time, and considering Equations (14), (19) and (24), we obtain

(27) $$\begin{array}{cc} \dot{W}= & z^{T}\dot{z}+e_{\theta_{1}}^{T}{\dot{e}}_{\theta_{1}}+e_{\theta_{2}}^{T}{\dot{e}}_{\theta_{2}}+\chi e_{z}^{T}{\dot{e}}_{z}\\ = & -z^{T}\left(B\alpha +B\beta +B\gamma -A\right)z\\ \; & -\lambda_{1}e_{\theta_{1}}^{T}e_{\theta_{1}}+e_{\theta_{1}}^{T}\dot{P}P^{-1}e_{\theta_{1}}-\lambda_{2}e_{\theta_{2}}^{T}e_{\theta_{2}}+e_{\theta_{2}}^{T}\dot{P}P^{-1}e_{\theta_{2}}\\ \; & -\chi e_{z}^{T}\left(B\alpha -A\right)e_{z}+\chi e_{z}^{T}Be_{\theta_{1}}^{T}z+\chi e_{z}^{T}Be_{\theta_{2}}^{T}z\\ \leq & -z^{T}\left(B\alpha +B\beta +B\gamma -A\right)z\\ \; & -\lambda_{1}∥e_{\theta_{1}}{∥}^{2}+\rho ∥e_{\theta_{1}}{∥}^{2}-\lambda_{2}∥e_{\theta_{2}}{∥}^{2}+\rho {∥e_{\theta_{2}}∥}^{2}\\ \; & -\chi \pi ∥e_{z}{∥}^{2}+\chi \varphi ∥B∥∥e_{z}∥∥e_{\theta_{1}}∥+\chi \varphi ∥B∥∥e_{z}∥∥e_{\theta_{2}}∥\\ = & -z^{T}\left(B\alpha +B\beta +B\gamma -A\right)z\\ \; & -(\sqrt{\lambda_{1}-\rho }∥e_{\theta_{1}}∥-\sqrt{\frac{\chi \pi }{2}}∥e_{z}{∥)}^{2}\\ \; & -2\sqrt{\lambda_{1}-\rho }\sqrt{\frac{\chi \pi }{2}}∥e_{\theta_{1}}∥e_{z}∥∥+\chi \varphi B∥∥e_{z}∥∥e_{\theta_{1}}∥\\ \; & -(\sqrt{\lambda_{2}-\rho }∥e_{\theta_{2}}∥-\sqrt{\frac{\chi \pi }{2}}∥e_{z}{∥)}^{2}\\ \; & -2\sqrt{\lambda_{2}-\rho }\sqrt{\frac{\chi \pi }{2}}∥e_{\theta_{2}}∥e_{z}∥∥+\chi \varphi ∥B∥∥e_{z}∥∥e_{\theta_{2}}∥\\ \leq & -z^{T}\left(B\alpha +B\beta +B\gamma -A\right)z\\ \; & +(-2\sqrt{\lambda_{1}-\rho }\sqrt{\frac{\chi \pi }{2}}+\chi \varphi ∥B∥)∥e_{z}∥∥e_{\theta_{1}}∥\\ \; & +(-2\sqrt{\lambda_{2}-\rho }\sqrt{\frac{\chi \pi }{2}}+\chi \varphi ∥B∥)∥e_{z}∥∥e_{\theta_{2}}∥\\ \leq & 0 \end{array}$$

According to Equations (26) and (27), we have $\lim_{t\to \infty }∥z∥=0$, $\lim_{t\to \infty }∥e_{z}∥=0$. Therefore, z(t) is bounded and $\lim_{t\to \infty }∥{\dot{e}}_{z}∥=0$. According to Equation (27), we have $\lim_{t\to \infty }∥e_{\theta_{1}}∥=\;0$, $\lim_{t\to \infty }∥e_{\theta_{2}}∥=\;0$. Because of $e_{\theta_{1}}\;=\;{\hat{\theta }}_{1}-\theta_{1}\;=\;{\left(-\hat{\beta }\right)}^{T}\;-\;{\left(-\beta \right)}^{T}\;=\;\beta^{T}\;-\;{\hat{\beta }}^{T}$, $e_{\theta_{2}}\;=\;{\hat{\theta }}_{2}\;-\;\theta_{2}\;=\;{\left(-\hat{\gamma }\right)}^{T}\;-\;{\left(-\;\gamma \right)}^{T}=\gamma^{T}-{\hat{\gamma }}^{T}$, we can obtain $\lim_{t\to \infty }∥\beta^{T}-{\hat{\beta }}^{T}∥=0$, $\lim_{t\to \infty }∥\gamma^{T}-{\hat{\gamma }}^{T}∥=0$. β,γ are assumed to be bounded, since they are the feedback gains of the humans. Therefore, $\hat{\beta },\hat{\gamma }$ are also bounded. According to Equation (10a–c), $P_{1},P_{2}$ are also bounded. According to Equation (10d), $A_{r}$ is bounded. Therefore, P,α and *u* are bounded.

According to Equation (10e), we can calculate the estimated errors $e_{Q_{1}}={\hat{Q}}_{1}-Q_{1}$, $e_{Q_{2}}={\hat{Q}}_{2}-Q_{2}$. $e_{Q_{1}},e_{Q_{2}}$ are due to the errors $e_{P},e_{P_{1}},e_{P_{2}}$. Because $e_{P},e_{P_{1}},e_{P_{2}}$ converge to zero, we have $\lim_{t\to \infty }∥e_{Q_{1}}∥=0$, $\lim_{t\to \infty }∥e_{Q_{2}}∥=0$, that is $\lim_{t\to \infty }{\hat{Q}}_{1}=Q_{1}$, $\lim_{t\to \infty }{\hat{Q}}_{2}=Q_{2}$.

Multiplying Equation (10d) by ${\hat{z}}^{T}$ on the left side and by $\hat{z}$ on the right side, and considering Equation (13), we have

(28) $$\begin{array}{cc} 0= & {\hat{z}}^{T}Q\hat{z}+{\hat{z}}^{T}P_{r}BB^{T}P_{r}\hat{z}+{\hat{z}}^{T}P_{r}\dot{\hat{z}}\\ \; & +\hat{z}P_{r}{\dot{\hat{z}}}^{T}+{\hat{z}}^{T}P_{r}He_{z}+\hat{z}P_{r}He_{z}^{T}\\ \equiv & \hat{\sigma }. \end{array}$$

Considering $\lim_{t\to \infty }e_{z}=0$, $\lim_{t\to \infty }{\dot{e}}_{z}=0$, we can obtain

(29) $$\begin{array}{cc} \lim_{t\to \infty }\sigma \equiv & \lim_{t\to \infty }(z^{T}QZ+z^{T}P_{r}BB^{T}P_{r}z\\ \; & +z^{T}P_{r}\dot{z}+zP_{r}{\dot{z}}^{T})\\ = & 0. \end{array}$$

Similarly, we can obtain

(30) $$\begin{array}{cc} \lim_{t\to \infty }\sigma_{1}\equiv & \lim_{t\to \infty }(z^{T}Q_{1}Z+z^{T}P_{1}BB^{T}P_{1}z\\ \; & +z^{T}P_{1}\dot{z}+zP_{1}{\dot{z}}^{T})\\ = & 0\\ \lim_{t\to \infty }\sigma_{2}\equiv & \lim_{t\to \infty }(z^{T}Q_{2}Z+z^{T}P_{2}BB^{T}P_{2}z\\ \; & +z^{T}P_{2}\dot{z}+zP_{2}{\dot{z}}^{T})\\ = & 0. \end{array}$$

$\lim_{t\to \infty }\sigma =0$, $\lim_{t\to \infty }\sigma_{1}=0$ and $\lim_{t\to \infty }\sigma_{2}=0$ indicate that the Nash equilibrium is achieved for the human-robot-human interaction system. ☐

## 4. Simulations and Results

### 4.1. Experimental Design and Ssimulation Settings

**Figure 3:**
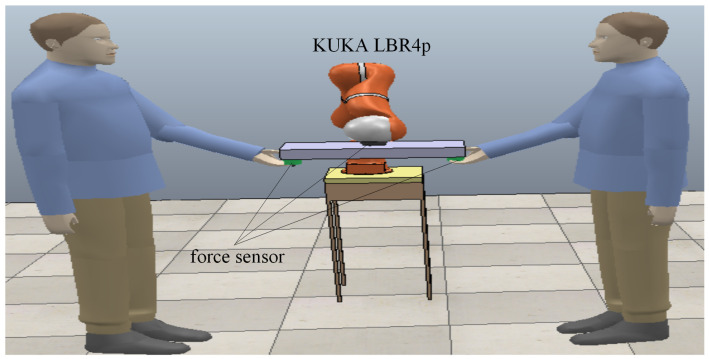
Simulation of cooperative object transporting task. The humans cooperate with the robot to transport the object back and forth between −10 cm and +10 cm along the horizontal direction. The forces that are exerted by the humans on the object are measured by force sensors at the interaction point.

With the development of the robot technology, in the future, robots will enter our homes and become a member of family in our daily lives. In our daily lives, we often need to carry various objects. Some objects (e.g., objects with smaller size and lower weight) can be successfully carried by one human; some objects (e.g., objects with medium size and medium weight) need to be carried successfully by two humans; some objects (e.g., objects with larger size and higher weight) can be carried successfully by three or more humans. Consider one scenario: In our home, we have an object (such as a table with a relatively larger size and higher weight) that need to be carried by three humans. However, there are only two humans in the home. In this case, we can let the robot help us carry the object together with the two humans. The robot can play the same role as one human. A simulation is conducted with CoppeliaSim in order to verify the control performance of the controller proposed in this paper. The version of CoppeliaSim that we used is CoppeliaSim 4.0.0 (CoppeliaSim Edu, Windows). Figure 3 demonstrates the CoppeliaSim simulation scenario of cooperative object transporting task. The humans cooperate with the robot to transport the object between −10 cm and +10 cm back and forth along the horizontal direction.

The controller that is proposed in this paper implements interactive control because every agent considers the control of other partners. In order to present the advantages of the proposed controller, we compare the proposed controller with the linear quadratic regulators (LQR) optimal controller. The LQR controller can be obtained by setting $A_{r}=A$, $A_{1}=A$, $A_{2}=A$ in Equation (10d–f). The LQR controller allows each agent to form its own control input optimally, but it ignores the controls of other partners. Let $Q=Q_{1}=Q_{2}=diag\left(100,0\right)$.

The cost functions of the humans usually change during the physical human-robot-human interaction. The robot needs to identify the change and adaptively adjust its own cost function in order to complete the cooperative object transporting task. In order to verify the ability of the robot to adaptively interact with two humans when humans’ cost functions change, we simulated a scenario where the robot cooperated with the humans to perform an object transporting task. The task performance is achieved by setting the value of *C* in Equation (21). Let C=diag(300,0). The cost functions of the human 1 and the human 2 change randomly according to $Q_{1}=diag\left(50,0\right)+\rho \cdot diag\left(50,0\right),Q_{2}=diag\left(50,0\right)+\rho \cdot diag\left(50,0\right)$ (ρ is a uniformly distributed random number between [0,1]).

The human-robot-human cooperative object transporting task can be fulfilled with less effort with the proposed controller. In order to make this affirmation, we made a comparison with a human-robot cooperative object transporting task. In simulation of the human-robot-human cooperative object transporting task, we let $Q=Q_{1}=Q_{2}=diag\left(100,0\right)$. In simulation of the human-robot cooperative object transporting task, we let Q=diag(100,0), $Q_{1}=diag\left(100,0\right)$, $Q_{2}=diag\left(0,0\right)$.

We assume that the humans and the robot do not have prior knowledge of each other (thus, initially $\hat{\alpha }\equiv 0,\hat{\beta }\equiv 0,\hat{\gamma }\equiv 0$). The control input of the robot are generated by Equations (5), (10a–f), (13), (15), (18) and (20). The simulated interaction forces $f_{1},f_{2}$ of the human 1 and the human 2 are generated by a similar set of equations. The simulation time is 40 s. Let the inertia of the robot $M_{d}$ = 6 kg, the damping of the robot $C_{d}=-0.2\;N\cdot m^{-1}$[19], the real-time least squares algorithm forgetting factor $\lambda_{1}=\lambda_{2}=0.95$. Simulation time step is 0.005 s.

### 4.2. Results

**Figure 4:**
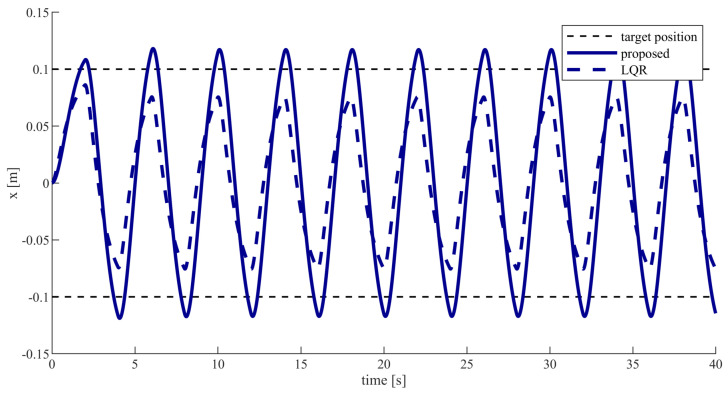
The end effector position value.

Figure 4 depicts the change in position of the end effector with respect to time. The results plotted in Figure 4 is a smooth curve that looks like a sinusoidal signal. This smooth curve is determined by Equation (3). In Equation (3), $u\left(t\right),f_{1}\left(t\right),f_{2}\left(t\right)$ are iteratively calculated by our proposed controller based on game theory. Due to the fact that the humans and the robot do not transport the object at a constant speed using our method, the end effector follows a curve signal rather than a straight line signal. As can be seen from Figure 4, the end effector can reach the target position with the proposed controller which means that the cooperative object transporting task is successfully fulfilled. In contrast, the end effector can not reach the target position with the LQR controller, which means that the cooperative object transporting task is not successfully fulfilled. The reason why the cooperative object transporting task can be successfully fulfilled with the proposed controller rather than with the LQR controller is that the proposed controller considers the interaction with other partners. When one partner decreases effort, the other partners will gradually increase their efforts to ensure the successful fulfillment of the cooperative object transporting task. In contrast, the LQR controller does not consider the interaction with other partners, so the cooperative object transporting task cannot be guaranteed to be successfully fulfilled.

**Figure 5:**
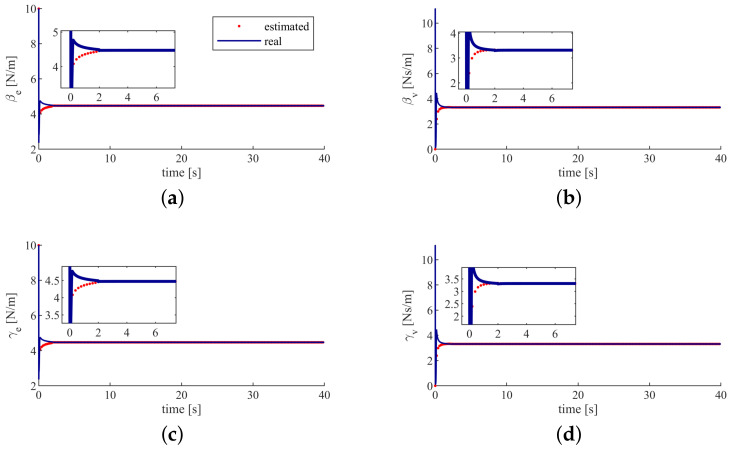
Control gains of humans. (**a**) the position error feedback gain of the human 1. (**b**) the velocity feedback gain of the human 1. (**c**) the position error feedback gain of the human 2. (**d**) the velocity feedback gain of the human 2.

In Figure 5, we can see that the estimated humans’ feedback gains converge to the real values in a few seconds. This means that the humans’ feedback gains can be successfully estimated by the proposed method.

**Figure 6:**
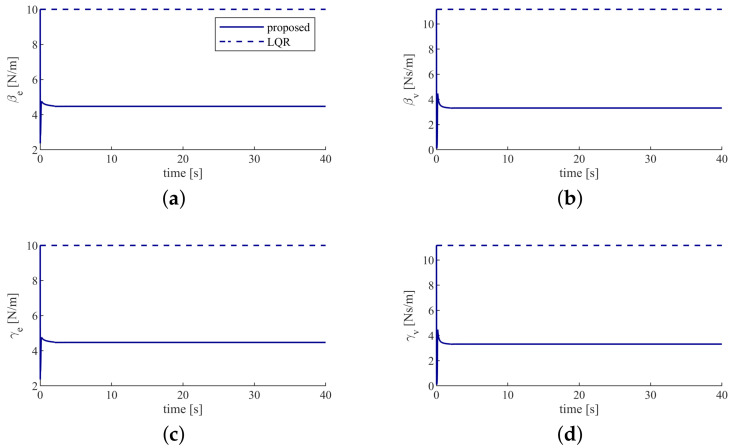
Humans’ control gains (**a**) the position error feedback gain of the human 1. (**b**) the velocity feedback gain of the human 1. (**c**) the position error feedback gain of the human 2. (**d**) the velocity feedback gain of the human 2.

Figure 6 demonstrates that fulfilling the cooperative object transporting task requires larger control gains β,γ with the LQR controller compared with the controller proposed in this paper. It means that accomplishing the same task requires less effort using the proposed controller. This is because that the proposed controller considers the interaction with other partners and calculates the minimal effort for the humans and the robot to complete the task. In contrast, the LQR controller doesn’t consider the interaction with other partners, so the humans and the robot only minimize their own cost function and may, therefore, require larger effort.

**Figure 7:**
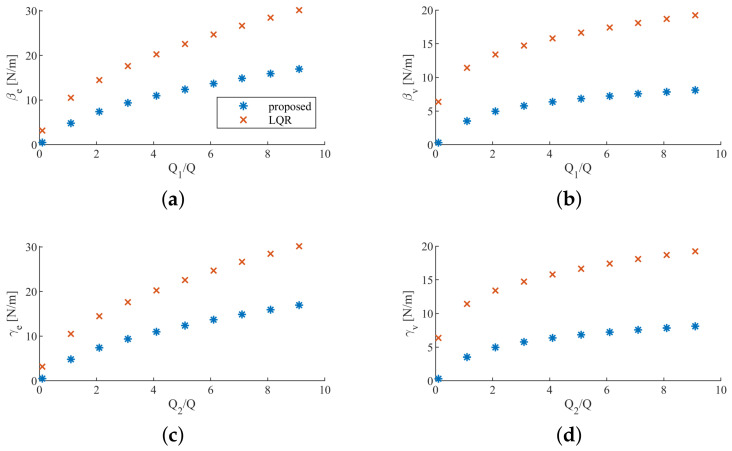
Control gains for different values of humans’ state weights. (**a**) and (**b**) the state weight of the human 1 vary. (**c**) and (**d**) the state weight of the human 2 vary.

The feedback gains are affected by the state weights of the cost functions. In order to verify the advantages of the proposed controller when the state weights vary, we let $Q_{1}$ vary from 0 to 10*Q* with $Q_{2}=diag\left(100,0\right)$ and let $Q_{2}$ vary from 0 to 10Q with $Q_{1}=diag\left(100,0\right)$ respectively. It can be seen from Figure 7 that accomplishing the same task always requires less effort using the proposed controller. We can also see that the difference between the control gains with our proposed controller and the control gains with LQR controller becoming smaller when $Q_{1}/Q$ or $Q_{2}/Q$ increases, this is because that the robot’s relative influence decreases.

From Figure 4, Figure 5, Figure 6 and Figure 7, we can conclude that the human-robot-human cooperative object transporting task can be fulfilled with less effort and the system can be kept stable using the proposed controller.

**Figure 8:**
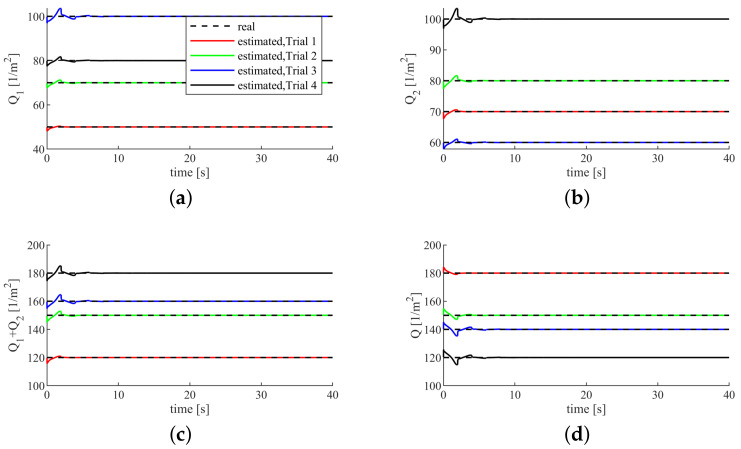
Humans’ state weights. (**a**) the state weight of the human 1. (**b**) the state weight of the human 2. (**c**) the sum of the state weights of the human 1 and human 2. (**d**) the state weight of the robot.

It can be seen from Figure 8 that, when the cost functions of the human 1 and the human 2 change, the cost function of the robot will also change adaptively. When the sum of the state weights of the human 1 and the human 2 $Q_{1}+Q_{2}$ increases, the state weight of the Robot *Q* decreases accordingly. Conversely, when the sum of the state weights of the human 1 and the human 2 $Q_{1}+Q_{2}$ decreases, the state weight of the robot *Q* increases accordingly. The reason why the robot can change adaptively is that we set the constant *C* value in Equation (21). Equation (21) makes the proposed controller able to adjust the contributions between the humans and the robot and makes the humans and the robot take complementary roles as well.

**Figure 9:**
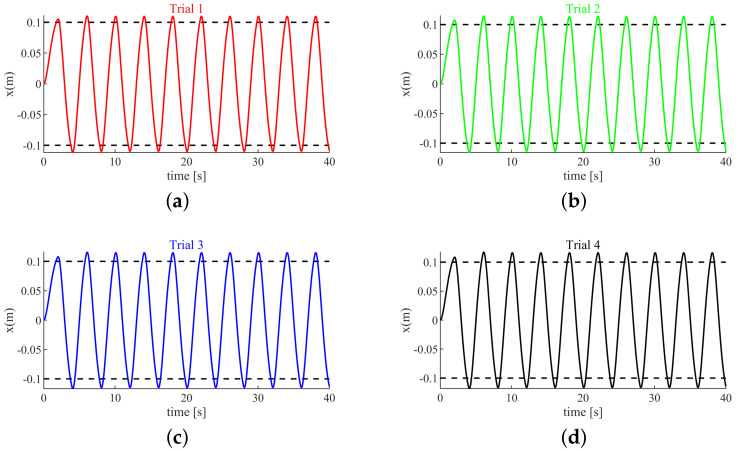
The end effector position value. (**a**) The end effector position value in Trial 1. (**b**) The end effector position value in Trial 2. (**c**) The end effector position value in Trial 3. (**d**) The end effector position value in Trial 4.

Figure 9 shows that, using the proposed controller, the adaptive cooperative object transporting task can be fulfilled and the system can be kept stable.

From Figure 8 and Figure 9, we can conclude that the adaptive cooperative object transporting task can be fulfilled with the proposed controller. During the physical interaction, the robot can successfully identify the change of each human’s cost function, and then adaptively adjust its own cost function to achieve interactive optimal control.

**Figure 10:**
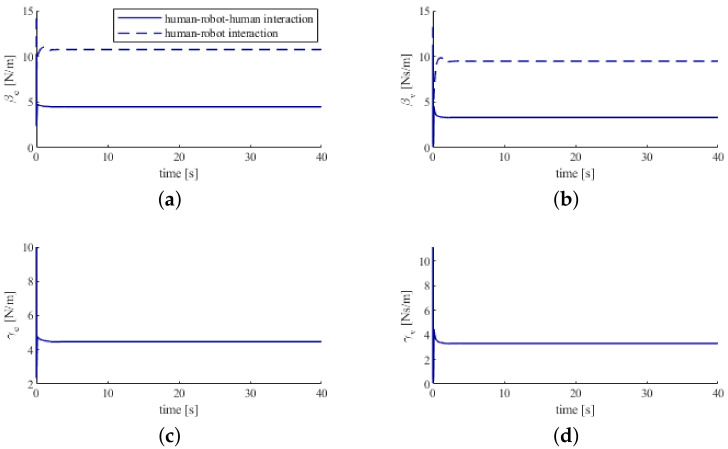
Humans’ control gains. The dashed lines correspond to the human-robot cooperative object transporting task. The solid lines correspond to the human-robot-human cooperative object transporting task. (**a**) the position error feedback gain of the human 1. (**b**) the velocity feedback gain of the human 1. (**c**) the position error feedback gain of the human 2. (**d**) the velocity feedback gain of the human 2.

Figure 10 demonstrates that fulfilling the human-robot-human cooperative object transporting task requires smaller control gains $\beta_{e},\beta_{v}$ as compared with the human-robot cooperative object transporting task. It means that accomplishing the same task requires less effort by means of the human-robot-human physical interaction. This is because the human-robot-human cooperative object transporting task considers the interaction with more partners (two partners) and calculates minimal effort for the humans and the robot to complete the task. In contrast, the human-robot cooperative object transporting task consider the interaction with less partners (only one partner), so the human and the robot may therefore require larger effort.

## 5. Conclusions

In this paper, the human-robot-human physical interaction problem has been studied. An adaptive optimal control framework for the human-robot-human physical interaction has been proposed based on N-player game theory. The recursive least squares algorithm based on forgetting factor has been used to identify unknown control parameters of the humans online. The performance of the controller proposed in this paper has been verified by simulations of cooperative object transporting task. The simulation results show that the proposed controller can achieve adaptive optimal control during the interaction between the robot and two humans and keep the system stable. Compared with the LQR controller, the proposed controller has more superior performance. Compared with the human-robot physical interaction, accomplishing the same cooperative object transporting task requires less effort by means of the human-robot-human physical interaction based on the approach proposed in the paper. Although this paper only conducts simulations on the physical interaction between one robot and two humans, it is worth mentioning that the framework that is proposed in this paper has the potential to be generalized to the situation where multiple robots physically interact with multiple humans. As future work, we will extend the framework to the interaction between multiple robots and multiple humans.

## References

[1] De Santis A., Siciliano B., De Luca A., Bicchi A. (2008). An atlas of physical human-robot interaction. Mech. Mach. Theory.

[2] Carolina P., Angelika P., Martin B. (2010). A survey of environment-, operator-, and task-adapted controllers for teleoperation systems. Mechatronics.

[3] Losey D.P., McDonald C.G., Battaglia E., O’Malley M.K. (2018). A review of intent detection, arbitration, and communication aspects of shared control for physical human-robot interaction. Appl. Mech. Rev..

[4] Aslam P., Jeha R. (2008). Safe physical human robot interaction-past, present and future. J. Mech. Sci. Technol..

[5] Li Y., Ge S.S. (2013). Human–robot collaboration based on motion intention estimation. IEEE-ASME Trans. Mechatron..

[6] Li Y., Ge S.S. (2016). Force tracking control for motion synchronization in human-robot collaboration. Robotica.

[7] Sandra H., Martin B. (2012). Human-oriented control for haptic teleoperation. Proc. IEEE.

[8] Chen Z., Huang F., Yang C., Yao B. (2019). Adaptive fuzzy backstepping control for stable nonlinear bilateral teleoperation manipulators with enhanced transparency performance. IEEE Trans. Ind. Electron..

[9] Liu C., Masayoshi T. Modeling and controller design of cooperative robots in workspace sharing human-robot assembly teams. Proceedings of the IROS 2014.

[10] Zanchettin A.M., Casalino A., Piroddi L., Rocco P. (2018). Prediction of human activity patterns for human-robot collaborative assembly tasks. IEEE Trans. Ind. Inform..

[11] Alexander M., Martin L., Ayse K., Metin S., Cagatay B., Sandra H. (2012). The role of roles: Physical cooperation between humans and robots. Int. J. Robot. Res..

[12] Costa M.J., Dieter C., Veronique L., Johannes C., El-Houssaine A. (2019). A structured methodology for the design of a human-robot collaborative assembly workplace. Int. J. Adv. Manuf. Technol..

[13] Daniel N., Jan K. (2019). A problem design and constraint modelling approach for collaborative assembly line planning. Robot. Comput. Integr. Manuf..

[14] Selma M., Sandra H. (2017). Control sharing in human-robot team interaction. Annu. Rev. Control.

[15] Mahdi K., Aude B. (2019). A dynamical system approach to task-adaptation in physical human-robot interaction. Auton. Robot..

[16] Roberto C., Vittorio S., Colombo R., Sanguineti V. (2018). Rehabilitation Robotics: Technology and Applications. Rehabilitation Robotics.

[17] Colgate J.E., Decker P.F., Klostermeyer S.H., Makhlin A., Meer D., Santos-Munne J., Peshkin M.A., Robie M. (2007). Methods and Apparatus for Manipulation of Heavy Payloads with Intelligent Assist Devices. U.S. Patent.

[18] Zoss A.B., Kazerooni H., Chu A. (2006). Biomechanical design of the Berkeley lower extremity exoskeleton (BLEEX). IEEE-ASME Trans. Mechatron..

[19] Li Y., Carboni G., Gonzalez F., Campolo D., Burdet E. (2019). Differential game theory for versatile physical human-robot interaction. Nat. Mach. Intell..

[20] Li Y., Tee K.P., Yan R., Chan W.L., Wu Y. (2016). A framework of human-robot coordination based on game theory and policy iteration. IEEE Trans. Robot..

[21] Nathanaël J., Themistoklis C., Etienne B. (2012). A framework to describe, analyze and generate interactive motor behaviors. PLoS ONE.

[22] Li Y., Tee K.P., Yan R., Chan W.L., Wu Y., Limbu D.K. Adaptive optimal control for coordination in physical human-robot interaction. Proceedings of the 2015 IEEE/RSJ International Conference on Intelligent Robots and Systems (IROS).

[23] Kirk D.E. (2004). Optimal control theory: An introduction. Optimal Control Theory.

[24] Li Y., Tee K.P., Chan W.L., Yan R., Chua Y., Limbu D.K. (2015). Continuous role adaptation for human-robot shared control. IEEE Trans. Robot..

[25] Lewis F.L., Vrabie D. (2009). Reinforcement learning and adaptive dynamic programming for feedback control. IEEE Circuits Syst. Mag..

[26] Vamvoudakis K.G., Lewis F.L. (2011). Multi-player non-zero-sum games: Online adaptive learning solution of coupled Hamilton–Jacobi equations. Automatica.

[27] Zhang H., Wei Q., Liu D. (2011). An iterative adaptive dynamic programming method for solving a class of nonlinear zero-sum differential games. Automatica.

[28] Liu D., Li H., Wang D. (2014). Online synchronous approximate optimal learning algorithm for multi-player non-zero-sum games with unknown dynamics. IEEE Trans. Syst. Man Cybern. Syst..

[29] Albaba B.M., Yildiz Y. (2019). Modeling cyber-physical human systems via an interplay between reinforcement learning and game theory. Annu. Rev. Control.

[30] Music S., Hirche S. Haptic Shared Control for Human-Robot Collaboration: A Game-Theoretical Approach. Proceedings of the 21st IFAC World Congress.

[31] Turnwald A., Wollherr D. (2019). Human-like motion planning based on game theoretic decision making. Int. J. Soc. Robot..

[32] Liu Z., Liu Q., Xu W., Zhou Z., Pham D.T. (2018). Human-robot collaborative manufacturing using cooperative game: Framework and implementation. Procedia CIRP.

[33] Bansal S., Xu J., Howard A., Isbell C. (2020). A Bayesian Framework for Nash Equilibrium Inference in Human-Robot Parallel Play. arXiv.

[34] Antonelli G., Chiaverini S., Marino A. A coordination strategy for multi-robot sampling of dynamic fields. Proceedings of the 2012 IEEE International Conference on Robotics and Automation.

[35] Yan Z., Jouandeau N., Cherif A.A. (2013). A survey and analysis of multi-robot coordination. Int. J. Adv. Robot. Syst..

[36] Martina L., Alessandro M., Stefano C. A distributed approach to human multi-robot physical interaction. Proceedings of the 2019 IEEE International Conference on Systems, Man and Cybernetics (SMC).

[37] Kim W., Marta L., Balatti P., Wu Y., Arash A. Towards ergonomic control of collaborative effort in multi-human mobile-robot teams. Proceedings of the IEEE/RSJ International Conference on Intelligent Robots and Systems.

[38] Starr A.W., Ho Y.-C. (1969). Nonzero-sum differential games. J. Optim. Theory Appl..

[39] Fudenberg D., Tirole J. (1989). Noncooperative game theory for industrial organization: An introduction and overview. Handb. Ind. Organ..

[40] Hegan N. (1985). Impedance Control: An Approach To Manipulation: Part I-Theory Part II-Implementation Part III-Applications. J. Dyn. Syst. Meas. Control.

[41] Blank A.A., Okamura A.M., Whitcomb L.L. (2014). Task-dependent impedance and implications for upper-limb prosthesis control. Int. J. Robot. Res..

[42] Vogel J., Haddadin S., Jarosiewicz B., Simeral J.D., Bacher D., Hochberg L.R., Donoghue J.P., van der Smagt P. (2015). An assistive decision-and-control architecture for force-sensitive hand–arm systems driven by human–machine interfaces. Int. J. Robot. Res..

[43] Basar T., Olsder G.J. (1999). Dynamic Noncooperative Game Theory.

[44] Shima T., Rasmussen S. (2009). UAV cooperative decision and control: Challenges and practical approaches. UAV Cooperative Decision and Control.

[45] Hudas G., Vamvoudakis K.G., Mikulski D., Lewis F.L. (2012). Online adaptive learning for team strategies in multi-agent systems. J. Def. Model. Simul..

[46] Tan H.J., Chan S.C., Lin J.Q., Sun X. (2019). A New Variable Forgetting Factor-Based Bias-Compensated RLS Algorithm for Identification of FIR Systems With Input Noise and Its Hardware Implementation. IEEE Trans. Circuits Syst. I Regul. Pap..

